# Implication of a new function of human tDNAs in chromatin organization

**DOI:** 10.1038/s41598-020-74499-7

**Published:** 2020-10-15

**Authors:** Yuki Iwasaki, Toshimichi Ikemura, Ken Kurokawa, Norihiro Okada

**Affiliations:** 1 Department of Bioscience, Nagahama Institute of Bio-Science and Technology, Nagahama, Shiga Japan; 2 Center for Information Biology, National Institute of Genetics, Mishima, Japan; 3 School of Pharmacy, Kitasato University, Sagamihara, Kanagawa Japan

**Keywords:** Transcriptional regulatory elements, Genome evolution

## Abstract

Transfer RNA genes (tDNAs) are essential genes that encode tRNAs in all species. To understand new functions of tDNAs, other than that of encoding tRNAs, we used ENCODE data to examine binding characteristics of transcription factors (TFs) for all tDNA regions (489 loci) in the human genome. We divided the tDNAs into three groups based on the number of TFs that bound to them. At the two extremes were tDNAs to which many TFs bound (Group 1) and those to which no TFs bound (Group 3). Several TFs involved in chromatin remodeling such as ATF3, EP300 and TBL1XR1 bound to almost all Group 1 tDNAs. Furthermore, almost all Group 1 tDNAs included DNase I hypersensitivity sites and may thus interact with other chromatin regions through their bound TFs, and they showed highly conserved synteny across tetrapods. In contrast, Group 3 tDNAs did not possess these characteristics. These data suggest the presence of a previously uncharacterized function of these tDNAs. We also examined binding of CTCF to tDNAs and their involvement in topologically associating domains (TADs) and lamina-associated domains (LADs), which suggest a new perspective on the evolution and function of tDNAs.

## Introduction

It is believed that tRNAs have been present since ancient times, prior to the separation of the three domains of life^1^. This suggests that tRNAs have some function other than their canonical function related to protein synthesis. In fact, the 3′ terminal nucleotides CCA of a tRNA-like structure serve as the initiation site for replication in RNA viruses such as Qβ-phage and turnip yellow mosaic virus^2, 3^. Based on this observation, Weiner and Maizels proposed the genomic tag hypothesis^2,3^, in which a tRNA first evolved as the recognition site for the initiation of replication. In addition, tRNA-derived fragments that are induced by stress exhibit various functions such as the inhibition of translation initiation, the ability to block apoptosis, the destabilization of various transcripts (including tumor-promoting transcripts) and the epigenetic inheritance of traits^4^.

Regarding the genes that encode tRNAs (tDNAs), which are multi-copy genes and are scattered throughout the genome of individual species, there is a correlation between codon usage and the number of individual tDNAs in prokaryotes^5^. Accordingly, it has been thought until recently that the function of tDNA is only to encode tRNA for its canonical function. In the case of eukaryotes, however, this relationship has not been observed^6^, implying that tDNAs have additional roles beyond protein synthesis. In fact, it is known that a DNA region harbouring tDNA has a conserved function as an insulator from yeast to humans^7^. For example, Raab et al. showed that certain human tDNA clusters, namely ALOX3 and the PER tDNA cluster, have insulator activities that include both enhancer and repressor blocking^7^. Several tDNAs are also involved in changes in chromatin structure^8–10^, as they are spatially clustered near the nucleolus although they are scattered throughout the genome in a linear sense. Furthermore, it has been shown that tDNAs are enriched at the boundary of topologically associating domains (TADs), which are self-interacting genomic regions and can mediate interactions between promoters and distant enhancers^11–15^. Thus, tDNAs may contribute to the demarcation of domain boundaries including TADs.

In a circular chromosome conformation capture analysis, it was suggested that tDNAs with insulator activity are involved in chromatin organization^7^. Generally, the insulator activity of the tDNAs is dependent on transcription factor III C (TFIIIC)^16–18^. In fission yeast, TFIIIC-binding loci may act as chromosome-organizing clamps by tethering distant loci to the nuclear periphery^19^. Recently, the function of TFs beyond their known regulation of gene expression has received much attention. Various TF binding sites (TFBSs) located in non-transcriptional regions have been observed, suggesting the presence of a novel functional significance of TFs such as affecting the functional concentration of various factors, induction of chromatin looping, changing chromatin and nuclear structure and the evolution of new transcriptional regulatory networks^20,21^. Among these functions, TF-mediated looping interactions between two different genomic regions has attracted wide attention^21^. We previously showed that TFBSs cluster in pericentric regions in the human genome^22–24^, providing an interesting possibility for the role of TFBSs in the association of pericentric DNAs of homologous and nonhomologous chromosomes^24,25^.

Various proteins other than polymerase III and TFIIIC are known to bind to tDNAs^26–28^, so tDNAs can serve as binding sites for various factors. For example, Pol II-associated factors, namely MYC, FOS and JUN and enhancer-binding proteins such as ETS1 and STAT1 bind near tDNAs, suggesting that these TFs are involved in activation of Pol III. Additionally, MYC recruits the histone acetyltransferase GCN5 and is involved in changes in chromatin structure. Interestingly, it was reported that CTCF also binds tDNAs^29^; CTCF is an insulator protein and is involved in the formation of domain structures including TADs and lamina-associated domains (LADs)^12,30–32^. In fission yeast, Fft3, a chromatin remodeling factor that is essential for maintaining chromatin structure at centromeres and subtelomeres, is enriched in tDNAs^33^. Thus, TFs that are involved in the formation of chromatin structure bind to tDNAs. Disruption of chromatin structure causes unusual gene regulation resulting in a disease phenotype^11,13,14^. Therefore, tDNAs and TFs (through their binding to tDNAs) should be involved in various mechanisms to regulate genes by controlling the chromatin structure.

It is important to examine the characteristics of TF binding to tDNAs to investigate functions associated with genomic structural changes including insulator activity. Although various studies have been carried out for tDNAs, comprehensive research has not been conducted. The present study systematically analysed the genomic profiles of tDNAs in human cell lines using the ENCODE data and found that many TFs bind to ~ 130 tDNAs. The TFs that bind to these tDNAs are thought to be involved in the formation of genomic special structures. Additionally, these tDNAs exhibit highly conserved synteny among tetrapods, suggesting that these tDNAs have played important roles during evolution. This is, to the best of our knowledge, the first report of a systematic analysis of colocalization of TFs on tDNAs and of the relationship between tDNA function and synteny.

## Results and discussion

### Characterization of the colocalization of TFs with tDNAs

As described above, not only the RNA Pol III complex but also the RNA Pol II complex and various TFs bind near tDNA regions^26–28^. However, these earlier studies focused on only a limited number of TFs; no comprehensive analysis has been performed to date. Here we investigated (1) what kind of TFs bind to a tDNA and in its vicinity, and (2) how many of the 489 human tDNA regions show a tendency to bind many TFs.

For this analysis, we used comprehensive datasets of human TFBSs based on chromatin immunoprecipitation sequencing (ChIP-seq) experiments from the ENCODE at UCSC (161 factors, 91 cells). In most cell lines, only a few TFs have been characterized. Accordingly, we selected six human cell lines, A549, GM12878, H1-hESC, HepG2, HeLa-S3 and K562, with many known TFs. In the cell line A549, 24 different TFs bind to 366,854 loci; in the cell line GM12878, 76 different TFs bind to 1,044,223 loci; in the cell line H1-hESC, 50 different TFs bind to 561,586 loci; in the cell line HepG2, 59 different TFs bind to 961,118 loci; in the cell line HeLa-S3, 55 different TFs bind to 690,753 loci and in the cell line K562, 100 different TFs bind to 1,315,515 loci. Raab et al*.* showed that human tDNAs located in chromosome 17 have insulator activities by using an enhancer- or repressor-blocking assay^7^. To examine the TF distribution in these tDNA regions and its relationship with any insulator activity, we first investigated the landscape of TFs near those tDNA regions.

**Figure 1:**
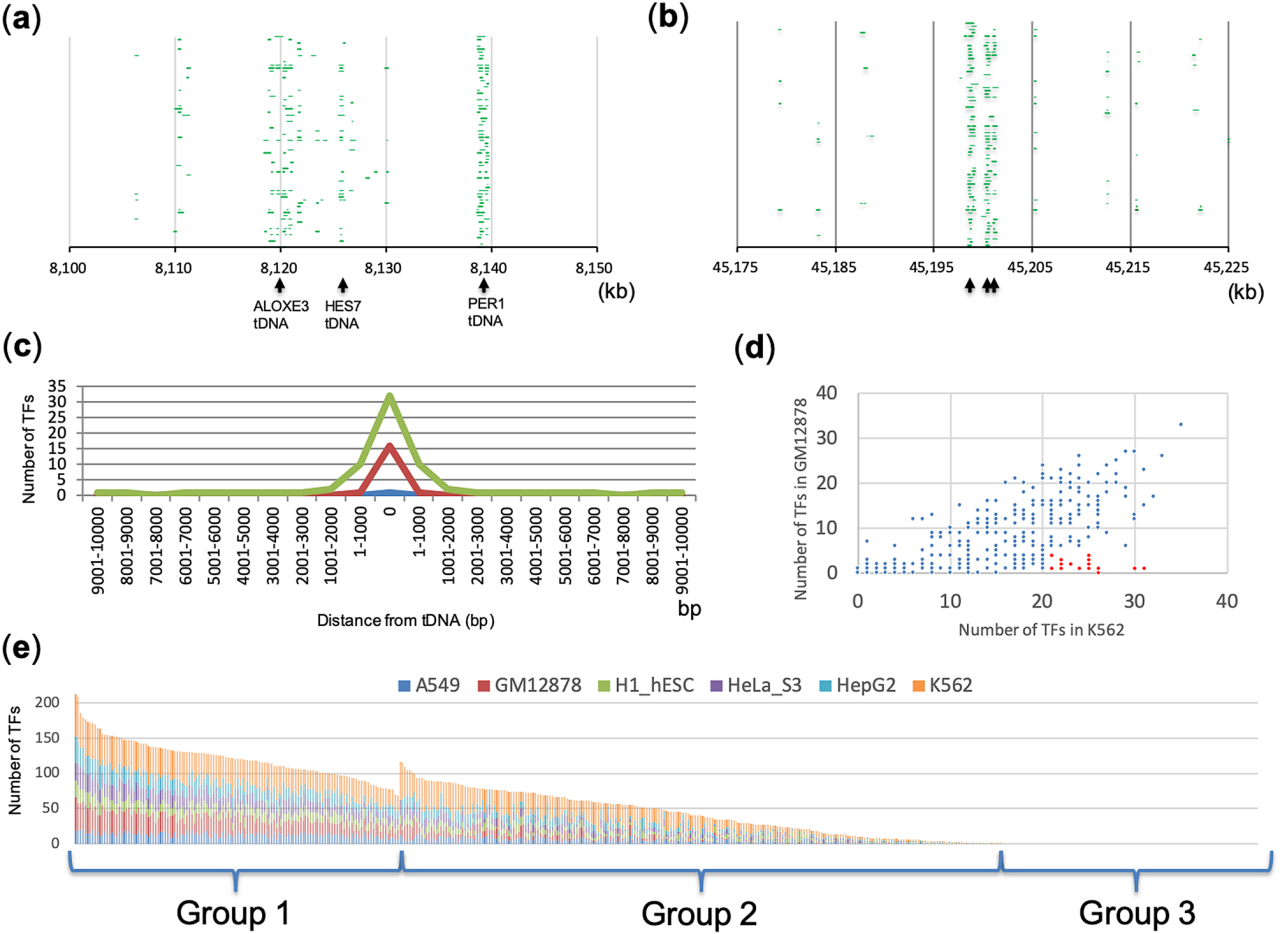
Many TFs bind to tDNA regions. Distribution of TFs at chr17:81,000,000**–**8,150,000 (**a**) and chr15:4,517,500**–**45,225,000 (**b**) in K562 cells. The regions bound by individual TFs are indicated with a green bar. (**c**) Distance between each tDNA and each TF binding region in K562. Upstream positions are shown to the left of 0 bp, and downstream positions are shown to the right; 0 bp indicates that the TF was bound to the tDNA. The 75th, 50th and 25th percentile values of the number of TFs bound to each position are indicated by green, red and blue lines, respectively. (**d**) The number of TFs bound to each tDNA in K562 and HeLa-S3 cells. tDNAs to which many TFs were bound in K562 cells but not GM12878 cells are shown as red data points. (**e**) The cumulative number of TFs bound to tDNAs in each cell line.

Figure 1a shows the distribution of TFs at chr17:81,000,000**−**8,150,000 in the cell line K562. This region has two tDNA clusters (the ALOXE3 tDNA cluster contains four tDNAs and the PER1 tDNA cluster contains two tDNAs) and one tDNA (HES7 tDNA), each of which is shown by an arrow in Fig. 1a. The tDNA cluster in ALOXE3 encodes tRNA-Lys-TTT-3-5, tRNA-Gln-CTG-1-5, tRNA-Leu-TAG-1-1 and tRNA-Arg-TCT-2-1 (Table 1 and Table S1). The tDNA cluster in PER1 encodes tRNA-Ser-CGA-1-1 and tRNA-Thr-AGT-5-1 (Table 1 and Table S1). The tDNA in HES7 encodes tRNA-Gly-GCC-2-6 (Table 1 and Table S1). Insulator activity was detected in the ALOXE3 and PER1 tDNA clusters but not in the HES7 tDNA^7^. Various TFs bind near the tDNA or tDNA clusters, but there were few TFs outside each of these three regions. Namely, 66, 74 and 19 TFs bound to the ALOXE3 tDNA cluster, the PER1 tDNA cluster and the HES7 tDNA region, respectively (Table 1). These data show a correlation between the number of TFs located in the tDNA region and its insulator activity. Despite the lack of insulator activity in HES7 tDNA region, TFIIIC bound to there as well as to both tDNA clusters, and tDNA-mediated insulator activity is known to depend on TFIIIC^16–18^. However, CTCF and YYI, also known as insulator proteins, were bound to the ALOXE3 and PER1 tDNA cluster but not to HES7 tDNA. Similarly, ARID3A and CHD2, which are involved in chromatin remodeling^34,35^, were bound to the ALOXE3 and PER1 tDNA cluster but not to the HES7 tDNA region. Accordingly, the number and composition of TFs seems to contribute to the insulator activity of a tDNA region by mediating configuration changes in chromatin structure. Next, we examined whether other tDNA regions also show a similar tendency. One example is shown in Fig. 1b (chr15:4,517,500–45,225,000). This region has three tDNAs, namely tRNA-His-GTG-1-7, tRNA-His-GTG-1-8 and tRNA-His-GTG-1-9, to which 42, 45 and 25 TFs were bound, respectively (Table 1). Interestingly, TFIIIC and YY1 were bound to all three tDNA regions, whereas CTCF, ARID3A and CHD2 were bound to tRNA-His-GTG-1-7 and tRNA-His-GTG-1-8 but not to tRNA-His-GTG-1-9. Whether tRNA-His-GTG-1-7 and tRNA-His-GTG-1-8 exhibit insulator activity is unknown, but a large number of TFs including insulator proteins were bound to these regions, as was the case for the ALOXE3 and PER1 tDNA clusters.

**Table 1:** The human tDNAs and tDNA clusters analysed in this study.

Cluster	Locus	tDNA	Unique TFs	Total TFs
PER	chr17:8,138,880**-**8,139,525	tRNA-Ser-CGA-1-1, tRNA-Thr-AGT-5-1	59	74
ALOXE3	chr17:8,119,154-8,121,012	tRNA-Lys-TTT-3-5, tRNA-Gln-CTG-1-5, tRNA-Leu-TAG-1-1, tRNA-Arg-TCT-2-1	38	66
HES7	chr17:8,125,745-8,125,816	tRNA-Gly-GCC-2-6	19	19
-		tRNA-His-GTG-1-8	45	45
-		tRNA-His-GTG-1-7	42	42
-		tRNA-His-GTG-1-9	25	25

This discovery prompted us to investigate comprehensively the distribution of TFs in all tDNA regions of the human genome (489 regions). First, we measured the distance between each tDNA region and each TF binding site in K562 cells (Fig. 1c) and in HeLa-S3, GM12878, HepG2, H1-hESC and A549 cell lines (Fig. S1). After calculation of the distance, we counted the number of TFs and calculated quartiles in each direction upstream and downstream from the tRNA. In K562 cells, the 75th percentile value (green line in Fig. 1c) corresponded to 32 TFs at 0 bp (i.e., overlapped the tDNA region). Additionally, other cell lines also showed the same tendency: the 75th percentile value reached 9, 15, 10, 12 and 14 at 0 bp in A549, GM12878, H1-hESC, HeLa-S3 and HepG2, respectively (green line in Fig. S1). A strikingly abrupt transition was shown for each quartile value in all cell lines. This finding that many TFs were bound within a limited region was consistently observed for many tDNA regions. We hypothesized that various TFs are tethered within a limited region of tDNA and could form a huge complex.

Next, to investigate whether the number of TFs bound to each tDNA varies or is consistent across cell lines, we compared the number of TFs that bounding to each tDNA between a pair of cell lines (Fig. S2). For this comparison, we counted the number of TFs in each cell line that overlapped with a tDNA by at least 1 bp. tDNAs to which a large number of TFs were bound in K562 exhibited a roughly linear tendency (Fig. S2) with those of other cell lines, and a high positive correlation was shown for each pair (r = 0.73–0.81; Table S3). Next, to perform this comparison more precisely, we chose 51 TFs that were commonly shared between K562 and GM12878. As observed in the above comparisons, tDNAs to which a large number of TFs were bound in K562 also tended to exhibit a rough linearity in GM12878 (Fig. 1d), and the correlation value reached 0.75 in this comparison. However, there were several tDNAs to which many TFs were bound in K562 but not in GM12878 (Fig. 1d). For example, more than 20 TFs bound to each of 17 tDNAs in K562, whereas < 5 TFs bound to the same tDNA in GM12878 (red data points in Fig. 1d). These tDNAs (17 among 489 tDNA regions) may be involved in roles specific to K562 cells. As each of the six cell lines analysed here originated from a particular organ (refer to the “Material and methods” regarding their origins), such binding specificity may reflect the organ-specific characteristics of TF binding properties of tDNAs.

By comparing the number of TFs that bound to each tDNA between a pair of cell lines as described above, we recognized three types of tDNAs, namely (1) tDNAs with abundant TFs in both cell lines, (2) tDNAs without TFs in both cell lines, (3) and the remaining tDNAs, which do not belong to either of the former two.

Next, by extending this observation to all six cell lines, we investigated more detailed characteristics of tDNAs bound by TFs. Figure 1e shows the cumulative number of TFs bound to each tDNA in each cell line. On average, 5.9, 8.7, 6.2, 7.2, 8.1 and 18.6 TFs were bound to tDNAs in A549, GM12878, H1-hESC, HeLa-S3, HepG2 and K562, respectively (Table S2). In this chart, Group 1 tDNAs are those to which more than the average number of TFs were bound in all six cell lines. Group 3 tDNAs are those to which no TFs were bound in any of these cell lines. The remaining tDNAs were classified into Group 2. As a result, we obtained 134, 249 and 106 tDNAs of the Group 1, 2 and 3, respectively. The tDNAs of Group1 and Group 3 are homogeneous within their respective groups in terms of their TF-binding properties in any cell line, whereas the tDNAs of Group 2 showed cell-specific TF-binding properties. As we used only six cell lines to develop these categories, it is possible that the group assignment of a particular tDNA might be altered when the binding profiles of various TFs across more cell lines is examined in the future.

Next, we calculated the frequency of particular TFs bound to Group 1 tDNAs in each cell line (Table 2 and Table S4). In K562, TFIIIC (99.3%) was bound to almost all tDNAs of Group 1 (Table 2), and the components of TFIIIB, namely BDP1 (97.8%), TBP (99.3%) and BRF1 (87.3%), were also bound to almost all tDNAs of Group 1 (Table 2); TFIIIB and TFIIIC are important for the insulator activity of tDNAs^7^. Interestingly, insulator proteins, namely CTCF (40.3%) and YYI (64.9%) were bound to about half of the tDNAs of Group 1 (Table 2). Additionally, several TFs related to chromatin remodeling, namely ARID3A (44.0%), CHD2 (62.7%), ETS1 (94.0%), ATF3 (100%), EP300 (97.0%), TBL1XR1 (97.0%) and RCOR1 (82.1%), were bound to most of the tDNAs of Group 1 (Table 2)^34–41^. As for the ALOXE3 and PER1 tDNA clusters (Fig. 1a), these TFs were correlated with tDNA insulator activity. ARID3A belongs to the AT-rich interaction domain (ARID) family of DNA-binding proteins, members of which participate in chromatin remodeling^35^. CHD2 is a member of the chromodomain helicase DNA-binding (CHD) family. Members of this family have a chromodomain that is involved in the remodeling of chromatin structure^34^. ATF3 is a member of the CREB/ATF family of basic region leucine zipper transcription factors and is involved in chromatin remodeling^37^. This protein interacts with histone deacetylase and causes acetylation of histones, resulting in modification of the chromatin structure. EP300 is a histone acetyltransferase and affects chromatin structure by acetylation of H3K122^41^. TBL1XR1 is an essential component of the SMRT/N-CoR complex, which is involved in chromatin regulation^39^. Thus, as most Group 1 tDNAs were bound by the above TFs, they should be important for the formation of chromatin structure.

**Table 2:** TFs bound to the Group 1 tDNAs in cell line K562.

TF	%	TF	%
ATF3	100.0	IRF1	70.0
RPC155	100.0	TRIM28	69.0
TFIIIC	99.0	YY1	65.0
TBP	99.0	CEBPB	63.0
TEAD4	99.0	JUN	63.0
BDP1	98.0	CHD2	63.0
EP300	97.0	CBX3	61.0
TBL1XR1	97.0	RBBP5	55.0
ETS1	94.0	BHLHE40	53.0
MYC	92.0	ELF1	51.0
BRF1	87.0	STAT1	49.0
JUND	84.0	KDM5B	48.0
MAZ	84.0	ARID3A	44.0
POLR3G	82.0	ZNF143	44.0
RCOR1	82.0	BCLAF1	43.0
MAX	80.0	MXI1	43.0
RFX5	80.0	UBTF	43.0
POLR2A	78.0	CTCF	40.0
THAP1	71.0		

CTCF is a transcriptional regulator that has been evolutionarily conserved from fruit flies to humans and carries out multiple functions, including its activity as an insulator. Recently, CTCF has been shown to colocalize with cohesin, on which its insulator activity is dependent^42–48^. Therefore, we searched the ENCODE data for tDNAs in which CTCF and cohesin were colocalized in each of the six cell lines; RAD21 and SMC3 were used as cohesin subunits in this analysis. Among these proteins, SMC3 had been noted in the four cell lines GM12878, HeLa-S3, HepG2 and K562, in all of which CTCF and RAD21 had also been observed. Accordingly, by using the above four cell lines, we detected 17 tDNAs in which CTCF, RAD21 and SMC3 were colocalized in at least one of the four cell lines (Table S5). CTCF and cohesin contribute to the formation of topologically associating domains (TADs)^12,30,31^. TADs are self-interacting genomic regions that are involved in various processes, including development, by mediating interactions between promoters and distant enhancers^14^.

Among the above 17 tDNAs, 7 tDNAs were bound by CTCF and cohesin in all four cell lines. In contrast, four tDNAs, namely genes for tRNA-Arg-ACG-1-3, tRNA-Gly-TCC-1-1, tRNA-His-GTG-1-6 and tRNA-Ser-AGA-2-3, were bound by CTCF and cohesion in only one of the four cell lines. For example, tRNA-Arg-ACG-1-3 was bound by these proteins only in HepG2 cells, tRNA-His-GTG-1-6 and tRNA-Gly-TCC-1-1 were bound by these proteins only in HeLa-S3 cells and tRNA-Ser-AGA-2-3 was bound by these proteins only in K562 cells, suggesting that these tDNAs have a specific function in each of these three cell lines. The remaining six tDNAs were bound by CTCF and cohesin in two or three cell lines. For example, tRNA-Asn-GTT-1-1 was bound by these proteins in HeLa-S3 and K562 cells; tRNA-Pro-AGG-2-1 was bound by these proteins in GM12878, HeLa-S3 and K562 cells; tRNA-Pro-AGG-2-6 and tRNA-Ser-GCT-4-3 were bound by these proteins in GM12878 and HeLa-S3 cells and tRNA-iMet-CAT-1-8 was bound by these proteins in HeLa-S3, HepG2 and K562 cells. Based on these results, it is likely that these tDNAs can act as binding sites for CTCF and cohesin and may contribute to the diverse functions that CTCF and cohesin have.

**Figure 2:**
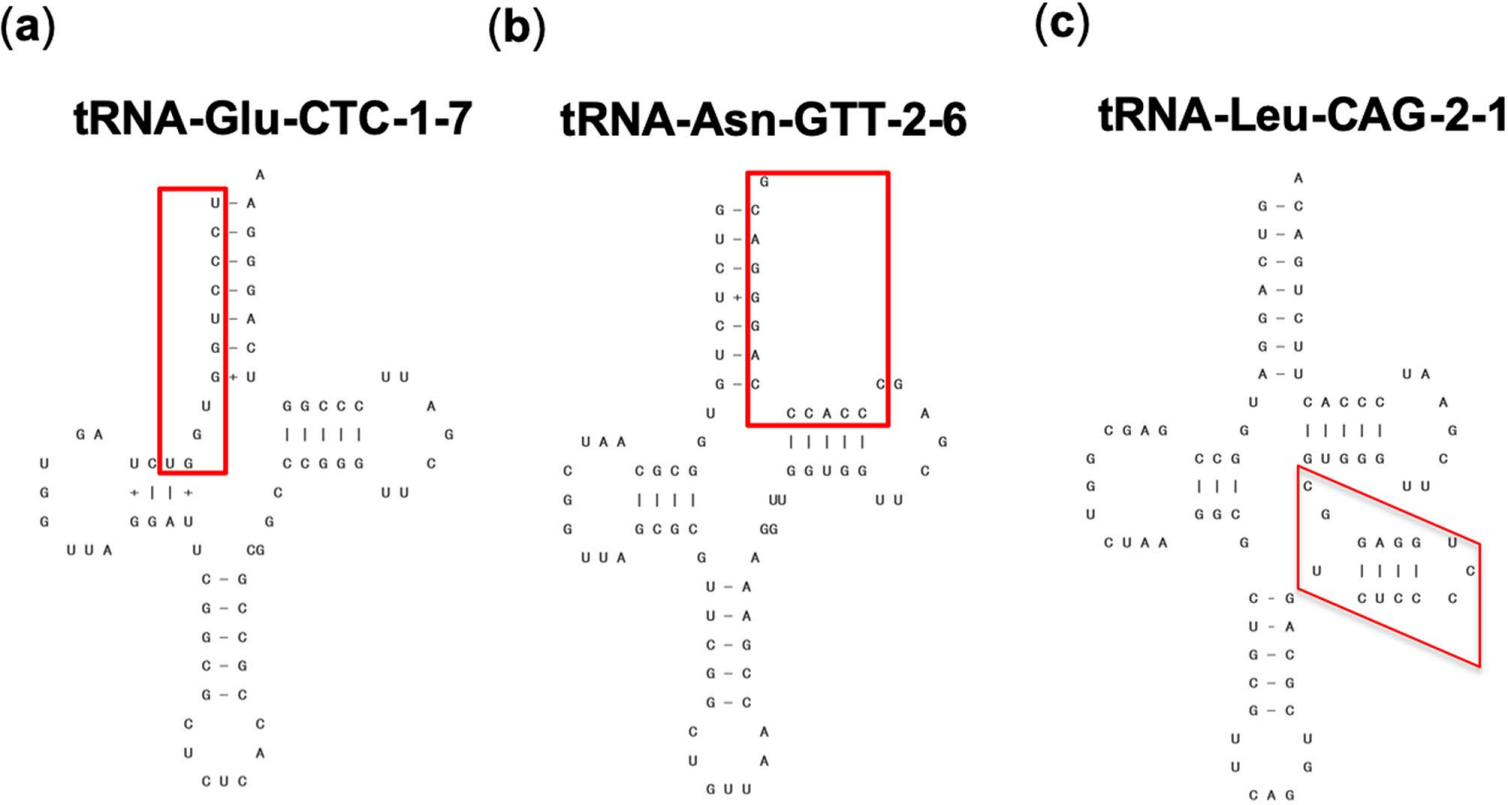
CTCF binding motifs are present in tDNAs. The two-dimensional structure of tDNAs tRNA-Glu-CTC-1-7 (**a**), tRNA-Asn-Gtt-2-6 (**b**) and tRNA-Leu-CAG-2-1 (**c**), where the CTCF binding motif was detected. The CTCF binding motif is represented by the red boxes.

CTCF uses different combinations of its zinc fingers to recognize divergent DNA sequences. Recent studies have identified core motifs to which CTCFs are bound and these were represented by position weight matrices (PWMs)^49^. Next, to examine the distribution of CTCF binding site in the tDNA sequences, we performed homology searches for the CTCF binding motifs on all 489 tDNA sequences using PWMs. We detected 129 tDNA sequences in which 147 motifs reside. Among these 147 motifs, 20 motifs overlapped with regions to which the identified TFs had been shown to bind, were included in the ENCODE data presented here (see the “Material and methods”) (Fig. 2, Table 3, Table S6). These 20 motifs were found in 16 tDNAs. There is striking congruence because this finding was consistent using two different methodologies, namely one from the analysis of sequence-based motifs and the other from ChIP-seq analyses. As the ENCODE data were obtained by using only six cell lines, it is likely that the remaining 127 CTCF motifs are bound to certain tDNAs in other cell lines. Accordingly, the ratio (20/147 = 0.136) may represent the ratio of six cell types analyzed here among all the possible cell type specificities. Among the 20 motifs, 7 motifs were detected between the 5′ terminal and D-loop, 7 motifs were detected between the T-loop and 3′ terminal, 5 motifs were detected between the anticodon loop and T-loop (which includes the variable loop) and the remaining 1 motif was detected between the D-loop and anticodon loop. Interestingly, the CTCF binding properties of these tDNAs were specific only for subsets of tDNAs in particular cell line(s) except for tRNA-SeC-TCA-1-1, to which CTCF bound in all six cell lines (Table S6). For example, tRNA-Leu-CAA-4-1, in which the CTCF binding motif was detected, was bound by CTCF in A549, GM12878 and HeLa-S3 cells but not in the other three cell lines.

**Table 3:** CTCF binding motifs detected in Group 1 tDNAs.

tDNA	Motif sequence (5′–3′)	Strand	Score
tRNA-Cys-GCA-2-3	CCCCCTCTGCCGGC	p	12.9052
tRNA-Arg-TCT-1-1	AGCCACCTGTTGGA	n	10.056
tRNA-Arg-CCT-2-1	CGCGCCCCAGTGGC	n	9.05525
tRNA-Leu-CAG-2-1	TCTCCCCTGGAGGC	p	7.41812
tRNA-Trp-CCA-2-1	TGTGACCTCGTGGC	p	6.36065
tRNA-iMet-CAT-1-6	TGTCCCCTTCAGCT	p	5.96006
tRNA-Arg-TCT-1-1	CTCCAACAGGTGGC	p	5.72964
tRNA-Trp-CCA-2-1	CGCAACCTTCTGAT	n	5.37508
tRNA-Leu-CAA-4-1	CGCCTCCATCCGGA	n	5.22539
tRNA-Glu-CTC-1-7	AGTTCCCTGGTGGT	n	5.02143
tRNA-SeC-TCA-1-1	TTCCACCTTTCGGG	n	4.99827
tRNA-Arg-ACG-1-3	TGCCATCTCAGGGC	p	4.90309
tRNA-Leu-CAA-4-1	CTCCATCCGGAGAC	n	4.7661
tRNA-Glu-TTC-1-1	TTCACCCAGGTGGC	n	4.67791
tRNA-His-GTG-1-1	AACCACCTGCCGTG	n	3.75582
tRNA-Arg-TCT-1-1	GGACTTCTAGAGGC	p	3.38381
tRNA-Ala-TGC-4-1	CGGCATCTCCAGAC	p	3.24217
tRNA-Asn-GTT-1-1	CGTCCCTGGGTGGG	n	3.07971
tRNA-Asn-GTT-2-3	CGTCCCTGGGTGGG	p	3.07971
tRNA-Asn-GTT-2-6	CGTCCCTGGGTGGG	n	3.07971

Positive and negative strand are indicated by p and n, respectively.

Next, we counted the tDNAs to which CTCF was bound in at least one of the six cell lines using ENCODE and detected 94 tDNAs (65 and 29 tDNAs from Group 1 and 2, respectively). In contrast, based on a motif search analysis using PWMs, CTCF binding motifs were detected in only 17% (16/94 = 0.170) of the above 94 tDNAs. It is possible that the remaining 78 tDNAs to which CTCF bound in at least one of the six cell lines had a noncanonical CTCF binding motif. Additionally, among the 16 tDNAs in which CTCF binding motifs were detected, certain tDNAs, namely tRNA-Arg-ACG-1-3, tRNA-Asn-GTT-1-1 and tRNA-SeC-TCA-1-1, were also bound by cohesin (Table S5). These tDNAs are thus more likely to exhibit insulator activity than other tDNAs.

### tDNAs contribute to the formation of domain structure

**Figure 3:**
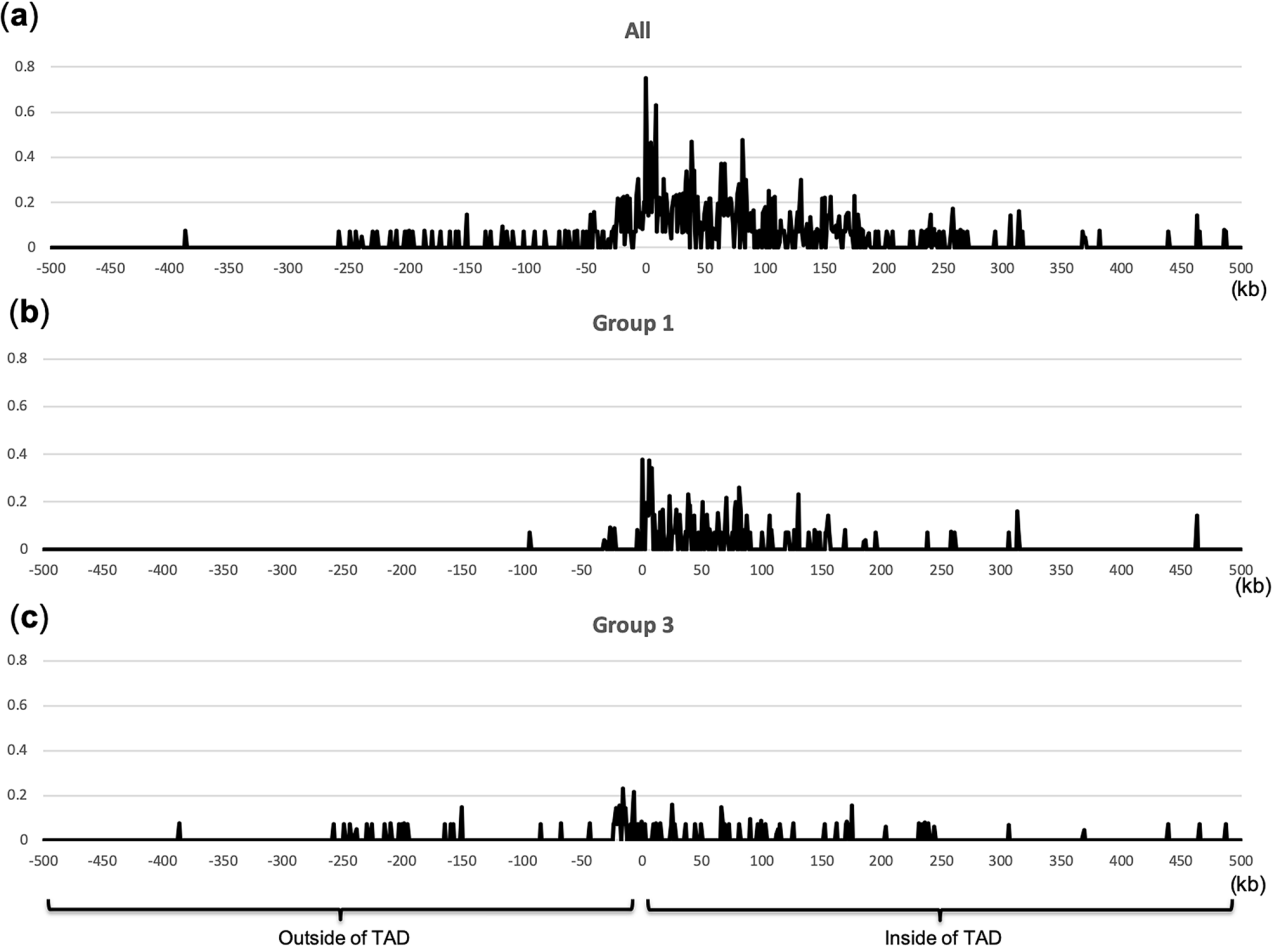
tDNAs are enriched in boundary regions of TADs. (**a–c**) Profiles of aligned TAD border regions are shown for all tDNAs (**a**), tDNAs from Group 1 (**b**) and tDNAs from Group 3 (**c**).

tDNAs are enriched at TAD boundaries^12^. TADs are found in eukaryote genomes and are important for the specific regulation of genes, as they mediate long-range enhancer-promoter interactions. The structure of TADs changes dynamically during cell cycle progression and is involved in various processes including cell cycle − specific gene regulation^48^. In general, the TAD structure mediates interactions between two elements located within the same domain. Disruption of this structure causes unusual gene regulation, resulting in pathogenic phenotypes^11–14^. For example, Symmons et al. showed that TAD structure mediates an interaction between *Shh* and its distal enhancer that is involved in the development of limbs and that disruption of the TAD structure results in abnormal limb development^14^. CTCF and cohesin are important for the formation of TAD structure^31^. Interestingly, the present study showed that 17 tDNAs (14 and 3 tDNAs from Group 1 and 2, respectively) are bound by CTCF and cohesin in at least one of the four cell lines, namely GM12878, HeLa-S3, HepG2 and K562 (see above, Table S5), raising the possibility that these tDNAs are also important for the formation of TADs. To look for a possible relationship between tDNAs and TADs, we measured the distance between each tDNA and each TAD border and discovered an enrichment of tDNAs near TAD borders (Fig. 3). Interestingly, tDNAs of Group 1, but not Group 3, tended to be localised at the border (Fig. 3b,c), suggesting that abundant TFs bound to such tDNAs demarcate some TADs by working together with CTCF and cohesin. We speculate that TFs bound to tDNAs contribute to the formation of TAD structures and regulate various functions that are controlled by TADs. Additionally, 92.5% (124/134 = 0.925) of tDNAs from Group 1 were located within the TAD, in contrast to only 49.1% (52/106 = 0.491) of tDNAs from Group 3. Two regions within TADs can freely interact with each other in a distance-independent manner^11–14^. Therefore, it is possible that, tDNAs from Group 1 can access other regions within the TADs via the TFs bound to them.

The nuclear lamina (NL) contributes to the spatial organization of chromosomes inside the nucleus^32,50^. Lamina-associated domains (LADs) are genomic regions that are in close contact with the nuclear lamina and account for more than one-third of the mouse and human genome. These regions correspond to heterochromatin and tend to exhibit low transcriptional activity, and they also overlap with domains of late DNA replication. This domain region helps to establish interphase chromosome topology and contributes to the overall spatial organization of the genome^50^. Therefore, insulator elements located at the boundaries of LADs may contribute to gene regulation and control of DNA replication timing by maintaining LAD structure. Guelen et al. discovered a striking enrichment of CTCF binding sites near LAD borders, which was greatest at ~ 5−10 kb outside the LADs^32^. Here, we measured the distance between each tDNA and its nearest LAD border and discovered two peaks of enrichment, ~ 0−30 and 65 kb outside the LADs (i.e., euchromatin region; Fig. S3a). Interestingly, the tDNAs from Group 1 were mainly found within 60−130 kb outside the LADs as well as around their boundaries (Fig. S3b), whereas the tDNAs from Group 3 were enriched at the boundary of LADs, suggesting that these two groups of tDNAs have different roles in the formation of LADs. Therefore, it is possible that these tDNAs are also involved in controlling gene regulation and DNA replication timing through maintaining LAD structure, in a manner similar to that suggested by the finding that CTCF binding is enriched at the boundaries of LADs.

### Accessibility and conservation of the tDNA region

**Figure 4:**
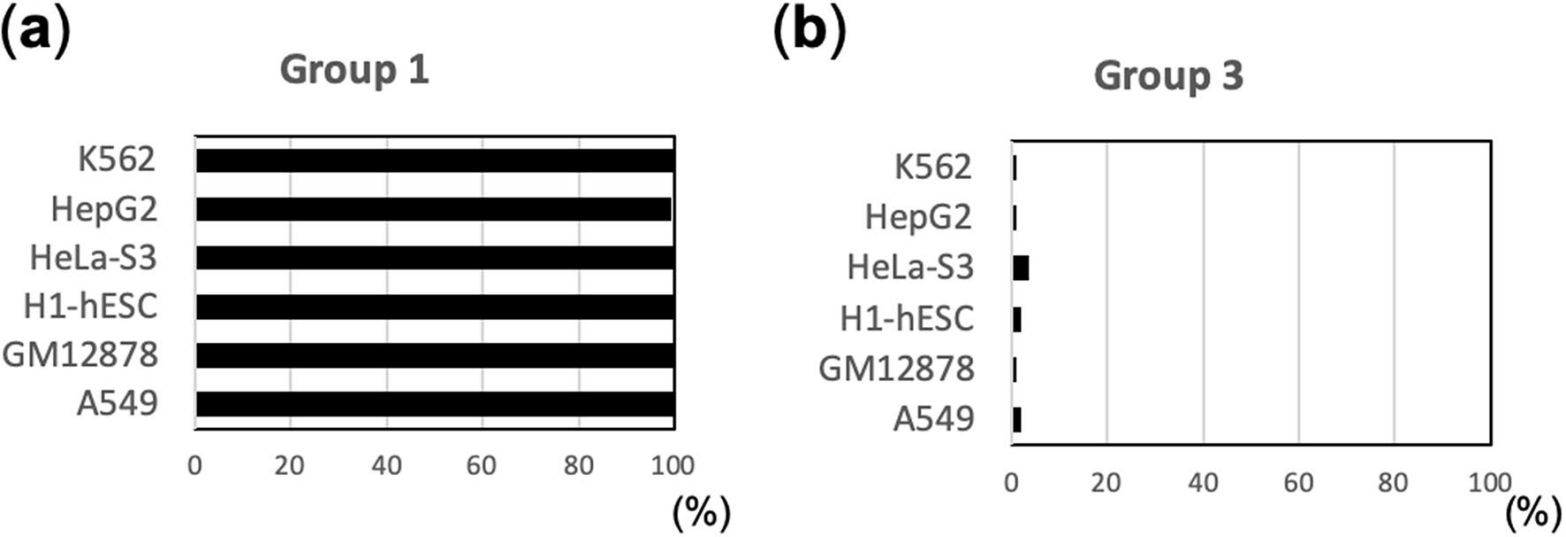
A high percentage of Group 1 tDNAs include DHSs. (**a**,**b**) Bar graphs show the percentage of tDNAs from Group 1 (**a**) and Group 3 (**b**) that include DHSs in each cell line.

Gene regulatory elements are associated with nucleosome-free regions that are known as DNase I hypersensitive sites (DHSs)^12, 51^. Therefore, we examined whether tDNA regions coincide with DHSs (Fig. 4). For this examination, tDNAs from Group 1 and 3 that overlapped with DHSs by at least 1 bp were counted in each of the six cell lines. In each cell line, almost all tDNAs from Group 1 included DHSs, whereas only a few tDNAs from Group 3 did so (Fig. 4). This result explains why various TFs can access tDNAs from Group 1 but not from Group 3.

**Figure 5:**
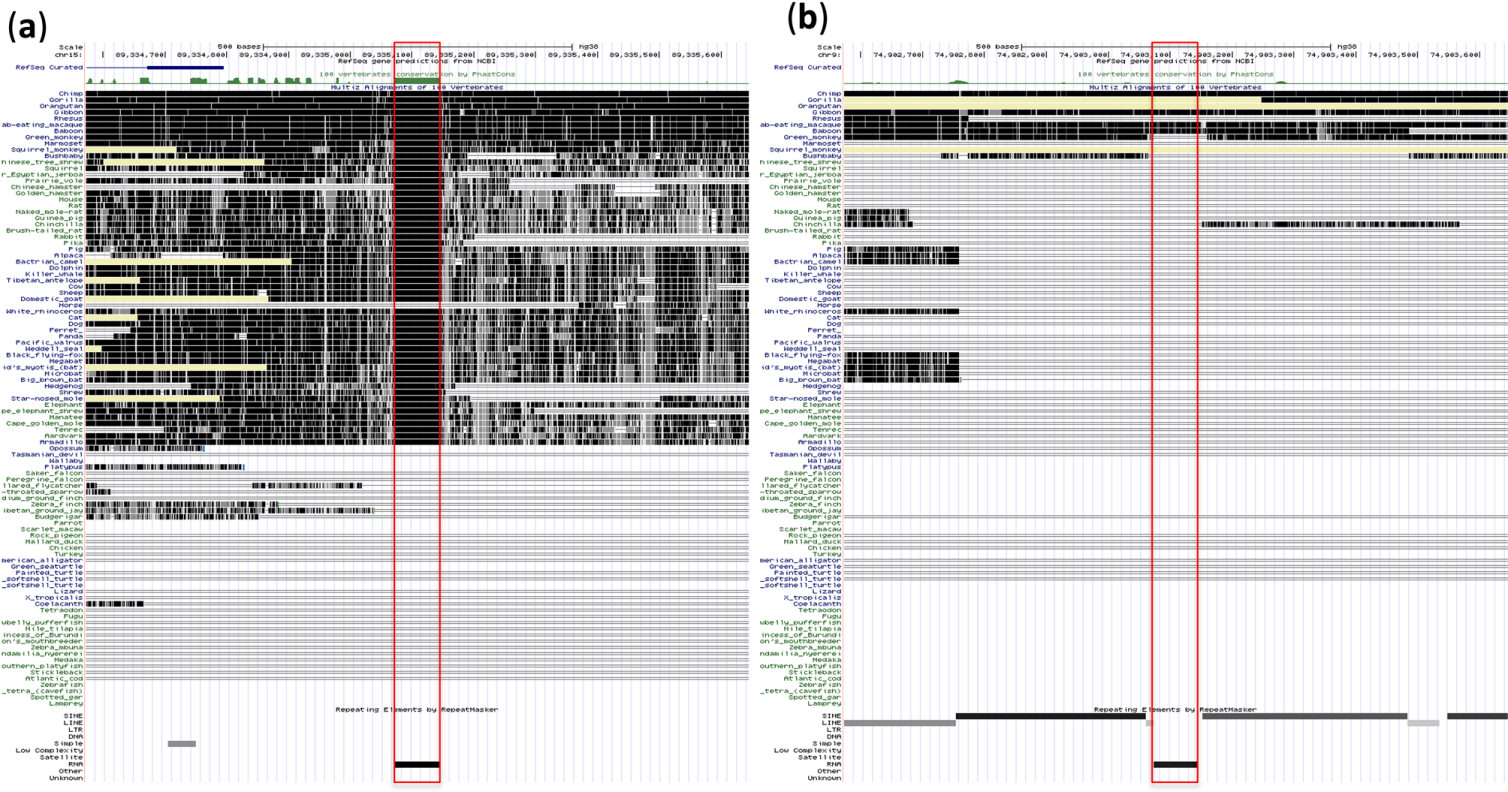
The tDNAs from Group 1 have been conserved during evolution. (**a**,**b**) Alignments of vertebrate genome sequences for the regions including the tDNAs for tRNA-Arg-TCG-1-1 (**a**) and tRNA-Asp-GTC-4-1 (**b**). tDNA region is indicated by red square. Aligned regions are indicated by black. Missing sequences in any assembly are indicated by yellow.

Conserved genomic regions are likely to have critical functional roles^52,53^. If tDNAs bound by abundant TFs including insulator proteins have important functions, these should be preserved across a wide range of organisms. Therefore, we investigated the possibility of whether these tDNA sequences have been conserved during the process of evolution. Figure 5a shows an example of a genome alignment using the UCSC Genome Browser, in which vertebrate genome sequences that include the tDNA for tRNA-Arg-TCG-1-1 (chr15:89,335,073–89,335,145), which belongs to Group 1, were aligned. This genomic region is conserved in many mammalian species ranging from humans to armadillos (Eutheria). As another example, vertebrate genome sequences that include the tDNA for tRNA-Asp-GTC-4-1 (chr9:74,903,074–74,903,145), which belongs to Group 3, are shown in Fig. 5B. This genomic region was conserved only from humans to baboons (primates). Across many tDNAs, we found the following tendencies: (1) the tDNAs belonging to Group 1 showed relatively conserved synteny among Eutheria or Theria, and (2) the tDNAs belonging to Group 3 showed synteny only among primates. Next, to confirm the above conclusion from a statistical point of view, we analysed the relative coincidence between conserved elements^52^, which were obtained from phastCons and are based on genome-wide multiple alignments with 99 vertebrate species, and tDNA regions. We quantified how many tDNAs overlapped with phastCons conserved elements by at least 1 bp. Among the tDNAs belonging to Group 1, 99% contained conserved elements. In addition to this result, in some tDNAs from Group 1, synteny was conserved (Fig. 5a and Fig. S4), which also suggests that they have played important roles other than their canonical function. Additionally, only 30% of the tDNAs from Group 3 contained conserved elements. Therefore, it is possible that the tDNAs from Group 3 may have been recently acquired during evolution.

In conclusion, tDNAs from Group 1 may contribute to the formation of domain structures such as TADs and LADs, and the high conservation of the synteny among tDNAs from Group 1 is consistent with this hypothesis. In contrast, tDNAs from Group 3 are conserved only among primates, and no TFs are bound to them. It is possible that tDNAs from Group 3 acquired primate-specific functions. The present work provides a means by which all tDNA data published to date can be re-evaluated with respect to the tDNA grouping described here and provides a foundation upon which future detailed studies can be built that characterize latent functions carried out by tDNAs and their bound TFs.

Finally, the aim of our study was to elucidate in a systematic fashion the whole picture of the function of tDNAs beyond their canonical function. Additionally, the findings obtained from this systematic analysis should be helpful and should provide basic information for scientists who study noncanonical tDNA functions.

## Material and methods

### Dataset

A total of 631 tDNA sequences and their bed file that contains annotations including chromosome information, start and end positions were obtained from GtRNAdb (https://gtrnadb.ucsc.edu/). Pseudo and mitochondrial tDNA loci were excluded from the dataset. Finally, we obtained 489 tDNA loci.

Bed files of ENCODE data (TF and DHS) and PhastCons Conserved Element were obtained from the UCSC. Among the TF bed files, we selected datasets from six human cell lines, namely A549, GM12878, H1-hESC, HepG2, HeLa-S3 and K562, which were derived from epithelial cells, B-lymphocytes, embryonic stem cells, a hepatocellular carcinoma, cervical carcinoma and leukaemia cells, respectively. We converted the genome coordinates from HG19 to HG38 using the LiftOver program available from UCSC (https://genome.ucsc.edu/cgi-bin/hgLiftOver). Among entries of TF data, a few that were > 1 kb were excluded, because they were possibly produced by experimental error. Moreover, it is not likely that one TF can bind to DNA spanning a > 1-kb region. Such regions made up < 1% of the total entries.

An annotation file of the TAD locations that were detected using the Directionality Index method was obtained from the Topologically Associating Domain Knowledge Base (https://dna.cs.miami.edu/TADKB/)^54^. We converted genome coordinates from HG19 to HG38 using the LiftOver program. A GFF file of LAD boundaries for the human genome was obtained from the Supplementary Data of Gulen et al*.*^32^. Again, we converted the genome coordinates associated with this file from HG18 to HG38 using LiftOver.

### CTCF binding motif

The CTCF binding motif was obtained from the human genome using the program STORM^55^. Then, the EMBL_M1 motif identified by Schmidt et al. was used for the PWMs^49^. Obtained motifs were filtered by the following criteria: (1) score was > 3.0, (2) the region where the motif was detected was consistent with ChIP-seq data (i.e., ENCODE data) and (3) the motif sequence overlapped with a tDNA sequence.

## Supplementary information

Supplementary file 1

Supplementary file 2
