# Supplementary material for: Implication of a new function of human tDNAs in chromatin organization
Source: Sci Rep. 2020 Oct 15;10:17440. doi: 10.1038/s41598-020-74499-7 (PMC7567086; doi:10.1038/s41598-020-74499-7)
Supplement: Supplementary file 2 [file 41598_2020_74499_MOESM2_ESM.docx]

**Implication of a new function of human tDNAs in chromatin organization**

Yuki Iwasaki^1,2^, Toshimichi Ikemura^1^, Ken Kurokawa^2^ and Norihiro Okada^1,3^*

*corresponding author: okadano@pharm.kitasato-u.ac.jp

^1^Department of Medical Biosciences, Nagahama Institute of Bio-Science and Technology, Nagahama, Shiga, Japan

^2^Center for Information Biology, National Institute of Genetics, Mishima, Japan

^3^School of Pharmacy, Kitasato University, Sagamihara, Kanagawa, Japan


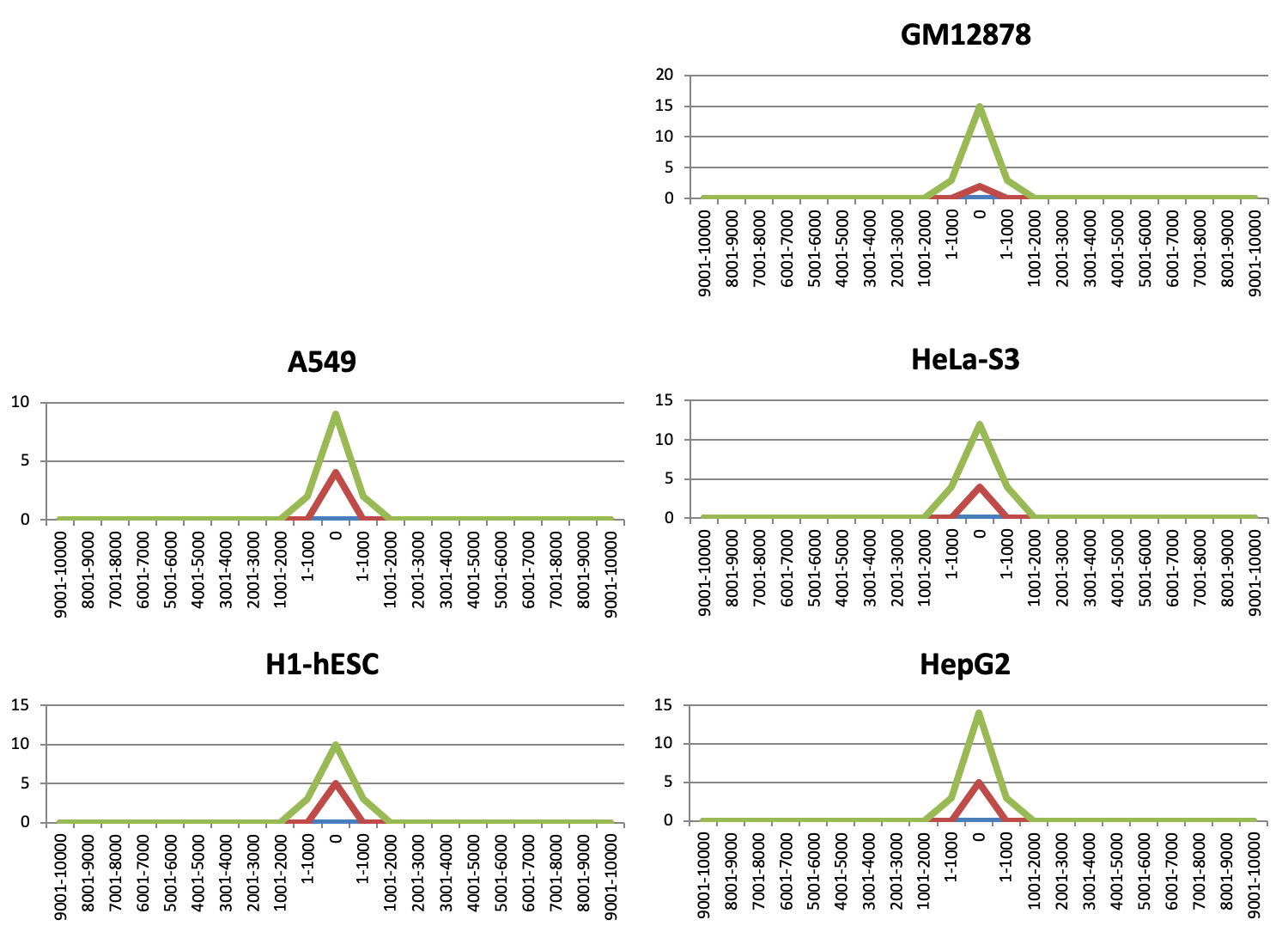


Figure S1. **Distance between each tDNA and each TF binding region in each cell line.** Upstream positions are shown to the left of 0 bp, and downstream positions are shown to the right; 0 bp indicates that the TF was bound to the tDNA. The 75^th^, 50^th^ and 25^th^ percentile values of the number of TFs bound to each position are indicated by green, red and blue lines, respectively.


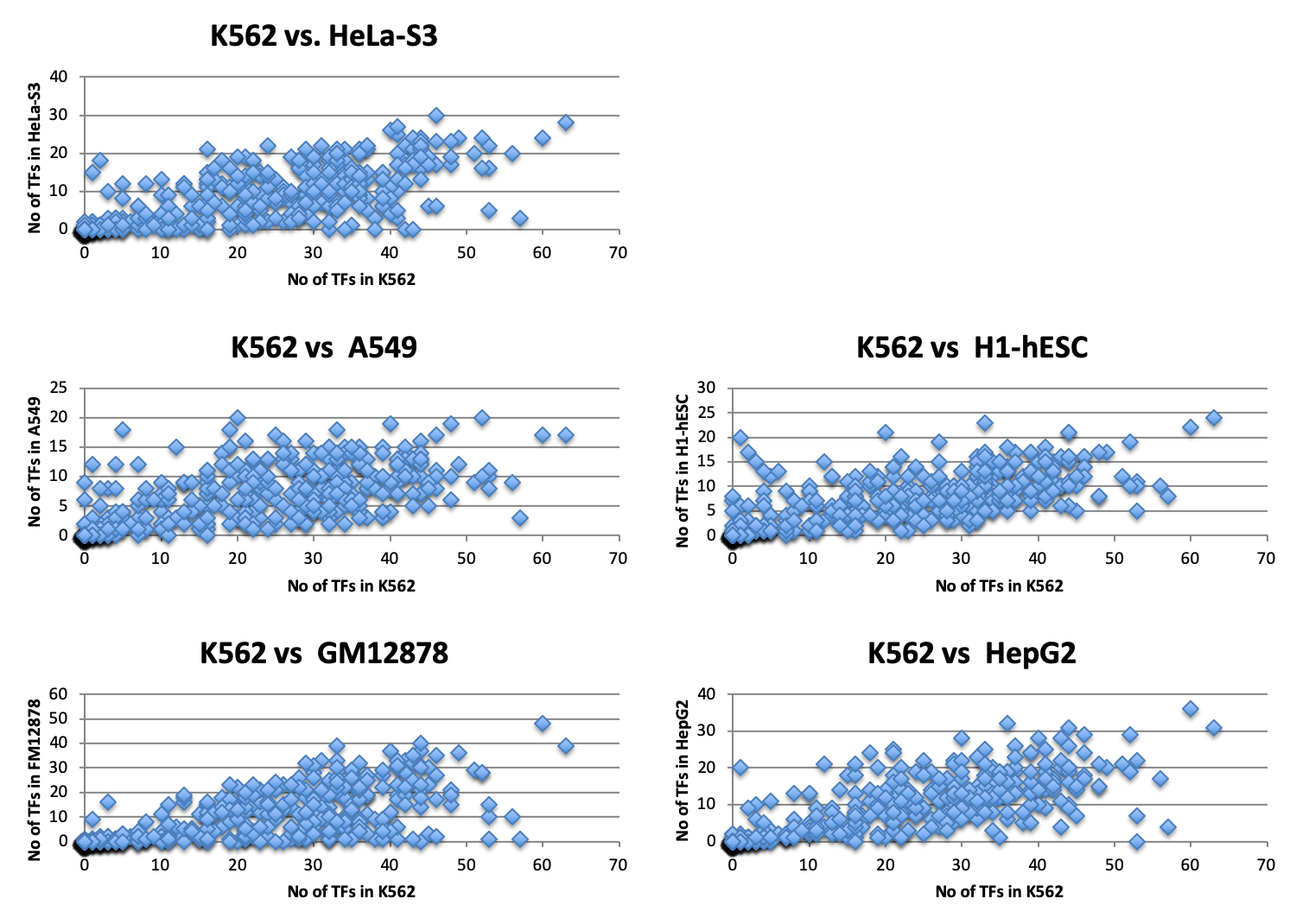


Figure S2. **The number of TFs bound to each tDNA between a pair of 2 cell lines.**


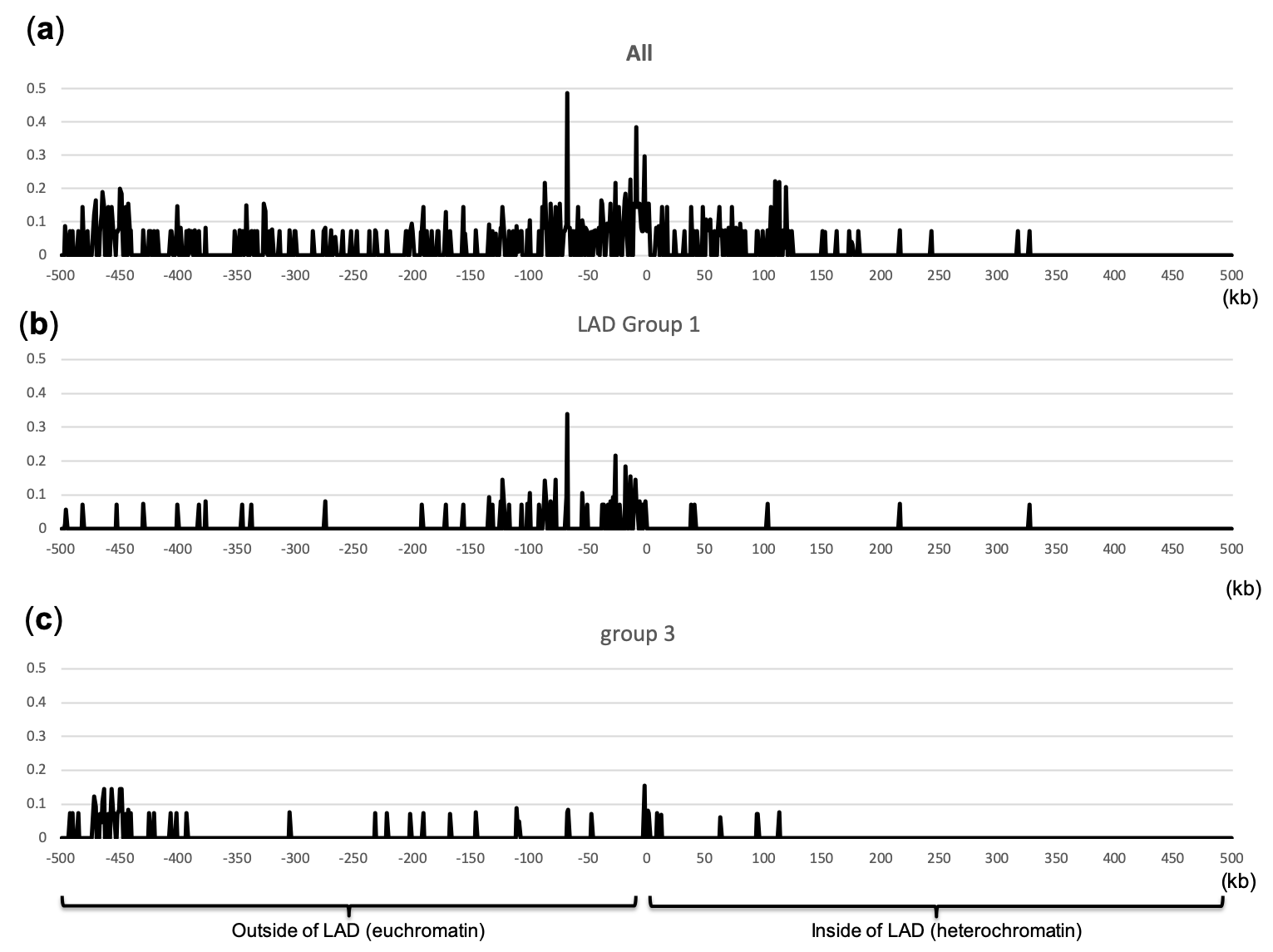


**Figure S3. tDNAs are enriched in boundary regions of LADs.** (a**−**c) Profiles of aligned LAD border regions are shown for all tDNAs (a), tDNAs from Group 1 (b) and tDNAs from Group 3 (c).


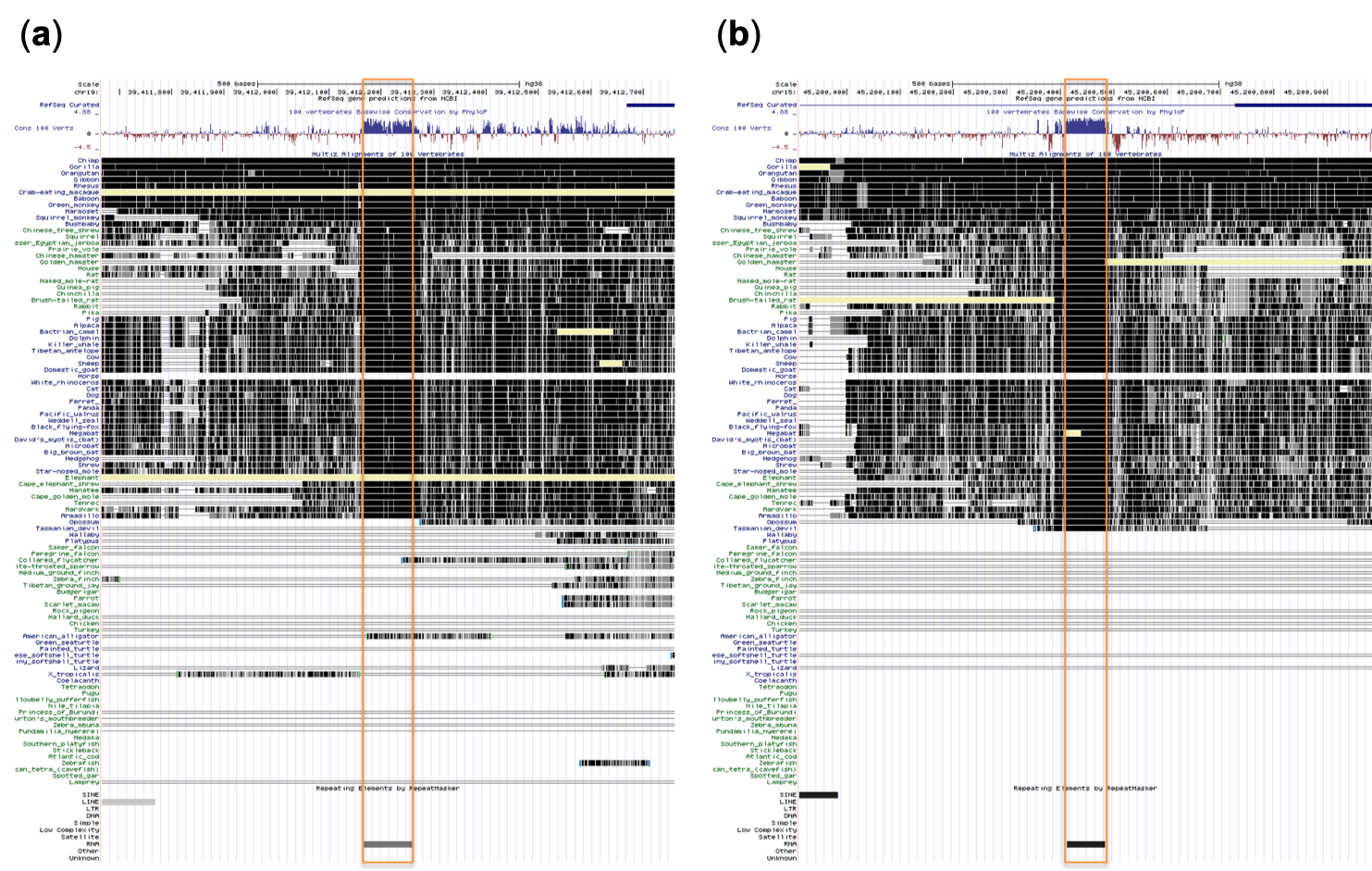


**Figure S4. The genome alignment of the vertebrate genome sequences including tDNA from Group 1**. tRNA-Ile-TAT-1-1 (a) and tRNA-His-GTG-1-8 (b).


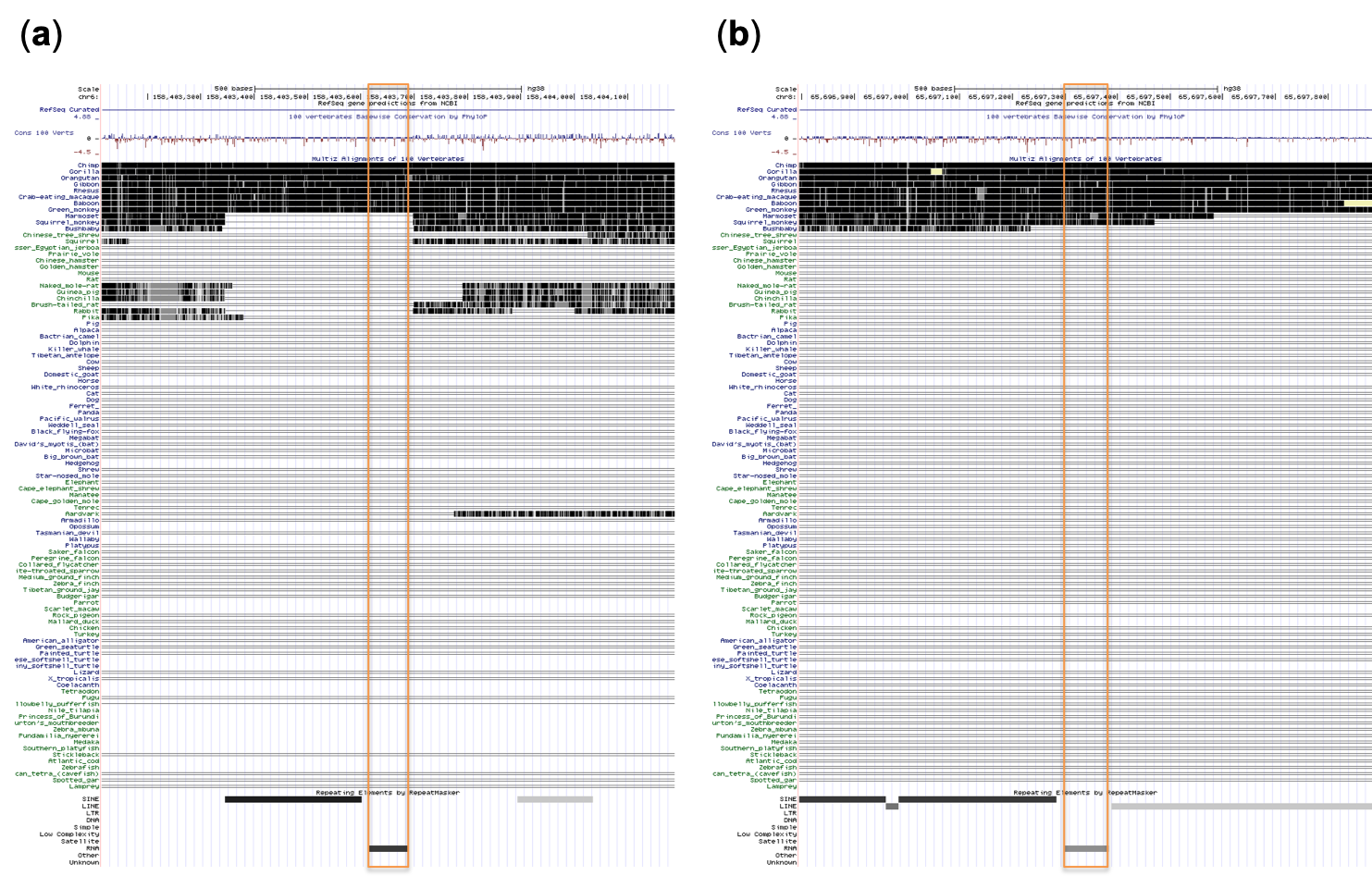


**Figure S5. The genome alignment of the vertebrate genome sequences including tDNA from Group 3**. tRNA-Val-AAC-chr6-87 (a) and tRNA-Tyr-GTA-9-1 (b).
